# Overcoming Dimensionality Constraints: A Gershgorin Circle Theorem-Based Feature Extraction for Weighted Laplacian Matrices in Computer Vision Applications

**DOI:** 10.3390/jimaging10050121

**Published:** 2024-05-15

**Authors:** Sahaj Anilbhai Patel, Abidin Yildirim

**Affiliations:** Department of Electrical and Computer, University of Alabama at Birmingham, Birmingham, AL 35205, USA; yildirim@uab.edu

**Keywords:** Gershgorin circle theorem, complex graph structure, dimensionality reduction, weighted Laplacian matrix, convolution neural network, feature extraction

## Abstract

In graph theory, the weighted Laplacian matrix is the most utilized technique to interpret the local and global properties of a complex graph structure within computer vision applications. However, with increasing graph nodes, the Laplacian matrix’s dimensionality also increases accordingly. Therefore, there is always the “curse of dimensionality”; In response to this challenge, this paper introduces a new approach to reducing the dimensionality of the weighted Laplacian matrix by utilizing the Gershgorin circle theorem by transforming the weighted Laplacian matrix into a strictly diagonal domain and then estimating rough eigenvalue inclusion of a matrix. The estimated inclusions are represented as reduced features, termed GC features; The proposed Gershgorin circle feature extraction (GCFE) method was evaluated using three publicly accessible computer vision datasets, varying image patch sizes, and three different graph types. The GCFE method was compared with eight distinct studies. The GCFE demonstrated a notable positive Z-score compared to other feature extraction methods such as I-PCA, kernel PCA, and spectral embedding. Specifically, it achieved an average Z-score of 6.953 with the 2D grid graph type and 4.473 with the pairwise graph type, particularly on the E_Balanced dataset. Furthermore, it was observed that while the accuracy of most major feature extraction methods declined with smaller image patch sizes, the GCFE maintained consistent accuracy across all tested image patch sizes. When the GCFE method was applied to the E_MNSIT dataset using the K-NN graph type, the GCFE method confirmed its consistent accuracy performance, evidenced by a low standard deviation (SD) of 0.305. This performance was notably lower compared to other methods like Isomap, which had an SD of 1.665, and LLE, which had an SD of 1.325; The GCFE outperformed most feature extraction methods in terms of classification accuracy and computational efficiency. The GCFE method also requires fewer training parameters for deep-learning models than the traditional weighted Laplacian method, establishing its potential for more effective and efficient feature extraction in computer vision tasks.

## 1. Introduction

Over the years, graph theory has expanded and gained significant advancements in various fields, such as chemistry, biology, and computer science [1,2,3]. Likewise, in machine learning, many problems can be modeled as a graph, where nodes represent pixels or regions, and edges describe relationships between nodes. The graph-based methods can capture and exploit an image’s spatial values and relational structures, offering a rich and flexible framework for image analysis and classification tasks [4]. Graph theory allows us to represent any graph in matrix form. The Laplacian matrix is one of the standard matrix forms used in graph representation. It conveniently represents a graph’s local and global properties. The Laplacian matrix can be formed in several ways; the most conventional matrix formation is by finding the adjacency matrix and its respective Degree matrix. Note that the Laplacian matrix grows larger in size with the increasing size of the image. This can lead to increasing computational time in postprocessing algorithms. Therefore, feature or dimensionality reduction is often a critical step when working with a large dataset. Additionally, it is vitally important to have a feature extraction algorithm that consumes less computational time.

In the past, the Laplacian Eigenmap (LE) was the most utilized nonlinear feature extraction method for the Laplacian matrix [5]. In LE, Belkin and Niyogi first compute the eigenvalues of the Laplacian matrix of a graph, and then, corresponding to their eigenvectors, the smallest non-zero eigenvalues are selected. In contracts to the LE method, He and Niyogi [6] proposed an algorithm called Locality Preserving Projections (LPP) that learns the linear mapping of data rather than a nonlinear mapping. Note that the LPP might not perform well on nonlinear structural data.

Besides calculating simple eigenvalues for feature extraction, Roweis and Saul [7] introduced Locally Linear Embedding (LLE), a manifold learning algorithm to project high-dimensional data into low-dimensional space. The fundamental principle of LLE involves selecting a predetermined number of nearest neighbors for each data point, typically referred to as the “k-number”. After identifying these neighbors, LLE calculates the local geometric structures by determining the best linear combination of these k-neighbors to reconstruct each data point. When transforming to a low-dimensional space, LLE ensures that these data points maintain their original proximities, staying as close together (or as far apart) as they were initially, preserving their relative distances and relationships. The drawback of LLE is that the user must define the “k-nearest neighbors” in it, which is not ideal for non-supervised operations. Moreover, the LLE is sensitive to noisy data and outliers.

The Isometric Feature Mapping (Isomap) [8] proposed by Tenenbaum et al. is another significant feature extraction method that finds the path with the shortest distance (also called geodesic distance) between all data point pairs in the local neighborhood. The geodesic distances help to capture the intrinsic manifold structure within the data. Similarly, He et al. [9] presented the “Laplacian score”, where the initial nearest neighbor graph is constructed and converted into a weighted Laplacian matrix. After that, the Laplacian score is calculated by deducting one from the feature variance and dividing by its degree, i.e., the number of connected nodes. Both LPP and LLE require a particular nearest neighbor graph and the Laplacian matrix.

Besides the feature extraction methods that are mentioned so far, several other feature extraction methods have been proposed that can be directly implemented on the Laplacian matrix. For instance, the Principal Component Analysis (PCA) [10] is the most commonly used linear feature extraction method in machine learning. In PCA, the data points are transformed orthogonally, and a new set of coordinates is generated, also known as principal components. The users select the number of principal components according to the data point’s total variance. However, increasing the number of data points increases the computation time for feature extraction. Another version of the PCA is called “kernel PCA” where the data points are mapped into higher dimensional space using the “kernel function”. After the data points are mapped, the principal components are computed. Then, as in standard PCA, the user selects the number of principal components according to the data point’s variance. Different types of kernel functions can be used for “kernel PCA”, such as the Radial Basis Function (RBF) [11] or the polynomial kernel function [12]. Note that the kernel PCA requires more computational time compared to the traditional PCA. Another alternative way to reduce computational time is by taking a smaller size of the dataset and reducing it to lower dimensions. Later, “dot-product” is used with the rest of the dataset to reduce the features. However, it might result in low classification performance. Another alternative way to reduce computational time is to reduce the dataset to smaller batches, such as Incremental PCA (I-PCA) [13], and then apply feature extraction techniques. However, it remains a critical step to determine the optimal batch size that balances computational efficiency with the enhancement of classification performance in feature reduction.

Additionally, addressing the computational efficiency in processing high-dimensional matrices remains a considerable challenge in developing feature extraction algorithms. The feature reduction methods reviewed in the preceding sections suggest an increase in computational demands proportional to the expansion of dataset sizes and dimensionalities, as exemplified by a dataset comprising 100,000 images, each with a resolution of 150 × 150 pixels. Motivated by this issue, the current study introduces an innovative approach to mitigate the ‘curse of dimensionality’ and low computational time without significantly compromising classification accuracy. This paper presents the development and application of a novel dimensionality reduction algorithm that surpasses various established feature extraction techniques in terms of classification accuracy while also demonstrating a noticeable decrease in computational time requirements. Furthermore, this research shows how the performance of these feature extraction algorithms is influenced by variations in image patch sizes.

The proposed algorithm utilizes the Gershgorin circle (GC) theorem for dimensionality reduction or feature extraction. The GC theorem was developed by mathematician S. A. Gershgorin [14] in 1931. The GC theorem estimates an eigenvalue inclusion of a given square matrix. The GC theorem has been used in several diverse applications, such as stability analysis of nonlinear systems [15], graph sampling in Graph theory [16], and evaluating the stability of power grids [17]. Over time, several extensions of the GC theorem have provided a better close estimation of eigenvalue inclusion of matrices [18,19]. The GC theorem is more time-efficient in computation than other eigenvalue inclusion methods [19]. However, none of the inclusion methods have been used for feature extraction tasks.

Once features are effectively extracted through any method, the subsequent pivotal step is to classify them by selecting an appropriate classification algorithm. The extracted features help not only to reduce computation time but also to reduce the number of training parameters that are required for the classification algorithms. In the fields of machine learning (ML) and deep learning (DL), many algorithms have been developed that provide state-of-the-art performance. In the field of ML, algorithms like Support Vector Machines (SVM) [20] and Decision Trees [21] are most commonly used, while in DL, algorithms such as artificial neural networks (ANN) [22] and convolution neural networks (CNN) [23] are some of the few algorithms that are commonly used.

**Figure 1:**
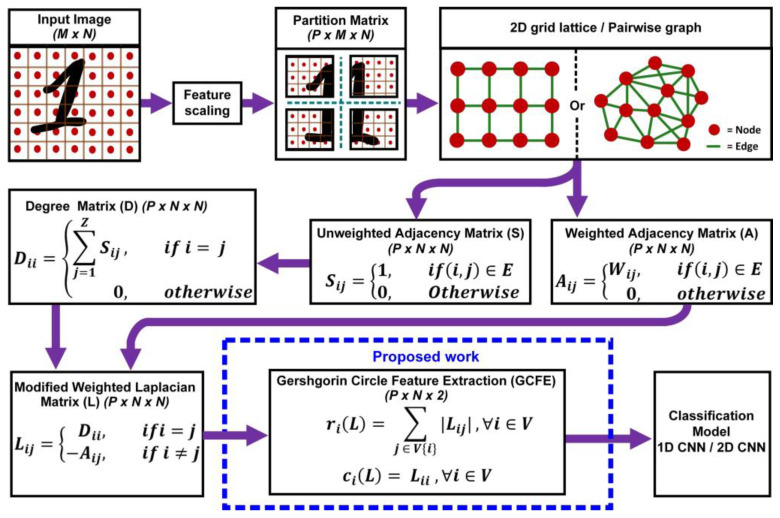
An overview of the GCFE methodology from image preprocessing to classification using the modified weighted Laplacian approach.

This paper introduces a novel feature extraction method for the graph-weighted Laplacian matrix by utilizing a mathematical theorem known as the Gershgorin circle theorem. Figure 1 shows the complete overview process of the proposed GCFE algorithm. The proposed algorithm modifies the weighted Laplacian matrix by converting it into a strictly diagonally dominant matrix termed a modified weighted Laplacian (MWL) matrix. Later, applying the GC theorem, the matrix’s P × N × N feature is reduced to P × N × 2 features, where P = no. of patches; N = no. of nodes, or total pixel size, accordingly. Finally, the reduced features are fed into the classification algorithm. For performance comparison, two classification algorithms, 1D-CNN and 2D-CNN, were utilized in this study. Detailed explanations of the proposed method, along with descriptions of the datasets used, are provided in Section 2. Section 3 discusses the results of the proposed methods, focusing on GCFE’s computational efficiency and performance accuracy compared to other feature extraction studies. This paper concludes with a summary of the findings and their implications in Section 4.

## 2. Materials and Methods

### 2.1. Datasets

This study utilizes three well-known and publicly available computer vision datasets with different image types, instances, and features. Table 1 presents the properties of each dataset.

#### 2.1.1. Extended MNIST (EMNSIT) Dataset

“The EMNIST” dataset is an extension of the original MNIST dataset that includes letters of the alphabet compared to the traditional digit classes. It was created by the National Institute of Standards and Technology (NIST) Special Database 19 [24]. The dataset includes seven sets, with digits, letters, and balanced and unbalanced sets, providing a variety of challenges for machine learning models. Each set has a 28 × 28 grayscale image with different numbers of classes and instances, as shown in Table 1.

#### 2.1.2. Cats vs. Dogs (CVD) Dataset

The “Cats vs. Dogs” dataset consists of 25,000 color images of 37 different breeds of dogs and cats. The dataset was created for the 2013 Kaggle competition [25]. All the images are resized to 100 × 100 × 3. While it has different objects in the background images, the target objects are in the foreground.

#### 2.1.3. Malaria Cell (MC) Dataset

The “malaria cell images” dataset was released by the National Institute of Health (NIH) [26], which consists of 27,558 instances equally divided between two classes. The dataset comprises parasitized and uninfected cells from segmented cells’ thin blood smear slide images. The dataset has RGB color images with a solid black color on the background. In our study, all images of the data sets were resized to 100 × 100 × 3 size.

### 2.2. Methodology

**Figure 2:**
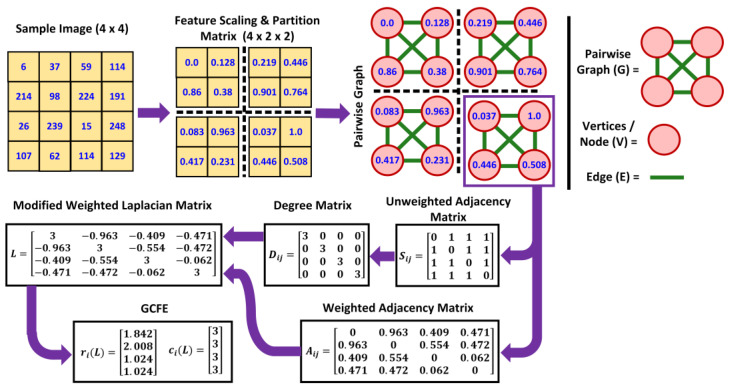
Flowchart of the proposed matrix transformation (pairwise graph) and GCFE for sample image size.

The proposed feature extraction algorithm consists of four principal steps—image preprocessing, formation of a MWL Matrix, GCFE, and classification. Figure 2 illustrates a detailed flowchart of GCFE formation with a sample image of 4 × 4 size.

#### 2.2.1. Preprocessing

The size of the input images was in M × N format, where M represents no. of row-pixels, and N represents no. of column-pixels, respectively. As shown in Figure 2, the sample image has M = 4 and N = 4. All the input image (pixel) intensities are initially scaled down from 0–255 to 0–1 by implementing min–max normalization, which is also known as feature scaling. After scaling, the input images are segmented into smaller patch (P) sizes. The images with smaller patches are called partition matrices with size P × M × N. The purpose of smaller patch sizes was to examine the performance of feature extraction algorithms on different patch sizes. The criteria for patch size selection were based on multiplying factors of the input image. For instance, if the input image size is 28 × 28, multiplying factors would be all numbers that they can divide evenly with the patch size, such as 2, 4, 7, 14, and 28, accordingly. For example, in Figure 2, the patch size = 2 for the sample image (i.e., 2 × 2); then, the 4 × 4 image is converted into a 4 × 2 × 2 partition matrix. In other words, the image will have 4 sub patches (P), each with 2 × 2 pixels. Similarly, if patch size = 28, the output partition matrix would be 1 × 28 × 28. The next step is converting each partition matrix into a graph (G). During image-to-graph conversion, image pixels are converted into a set of vertices or nodes (V) (represented by red circles in Figure 2). The connections between sets of nodes are called edges (E) (represented by green lines in Figure 2). For this operation, any graph conversion method can be used. In this study, three different graph conversion methods have been used. These are called “2D-grid lattice”, “pairwise graph”, and “K-nearest neighbors (K-NN) graph”, accordingly. The three graph methods are utilized to justify the performance of the proposed feature extraction on different graph structures. In Figure 2, each 2 × 2 partition matrix is converted to a graph using a pairwise graph. Each edge is weighted according to the “Manhattan distance” between any two given nodes. Equation (1) presents the formula to calculate the weighted edge ($W_{ij}$) of any given pair of nodes in a graph representation of an image.

(1) $$W_{ij}=\left|\left.{value}_{2}-{value}_{1}\right|\right.$$

where

$W_{ij}$ = weight of the edge between the node i^th^ and j^th^;

${value}_{1}$ = the pixel value at the coordinates (x_1_, y_1_) for i^th^ node inside image.

${value}_{2}$ = the pixel value at the coordinates (x_2_, y_2_) for j^th^ node inside image.

#### 2.2.2. Modified Weighted Laplacian (MWL) Matrix

Graphs are generally transformed into matrix forms to facilitate interpretation or processing. The most common way is the weighted or unweighted adjacency matrix. The weighted adjacency matrix (A) is the Z×Z square matrix, where Z represents the total number of nodes. The total number of nodes in matrix A is equal to the number of rows multiplied by the number of columns in the image patch size. The elements of the undirected graph weighted adjacency matrix are formed using Equation (2).

(2) $$A_{ij}=\left\{\begin{array}{c} W_{ij},\;\;if(i,j)\in E,\\ 0,\;\;otherwise, \end{array}\right.$$

where

$W_{ij}$ = weight of the edge between nodes i and j;

E = the set of edges in the graph such that (i,j) is an edge connecting node i and j;

$A_{ij}$ $=({i,j)}^{th}$ entry of the weighted adjacency matrix A.

In Figure 2, it can be depicted that the 4th patch of the sample image is converted into a 4 × 4 weighted adjacency matrix from a 2 × 2 partition matrix pairwise graph. Each entry in the matrix represents the weight of the edge according to Equation (1). Note that the matrix is symmetric for undirected graphs. To construct the modified Laplacian matrix, it is essential to compute the Degree matrix. Typically, the Degree matrix (D) is calculated by taking the row summation of the weighted adjacency matrix. Instead, we computed the elements of the unweighted adjacency matrix (S) using Equation (3). In unweighted adjacency, the matrix represents the presence or absence of edges between nodes in the graph. The entries of the S matrix are typically binary, where “1” indicates that there is an edge between nodes i and j, and “0” indicates that there is no edge between nodes i and j. Then, the Degree matrix (D) is computed as described in Equation (4), where each diagonal entry $D_{ii}$ represents the degree of the $i^{th}$ node.

(3) $$S_{ij}=\left\{\begin{array}{c} 1,\;\;if(i,j)\in E,\\ 0,\;\;otherwise, \end{array}\right.$$

where

$S_{ij}$ =$({i,j)}^{th}$ entry of the unweighted adjacency matrix S,

E = the set of edges in the graph, where (i,j) are edge-connecting nodes i and j.

(4) $$D_{ii}=\left\{\begin{array}{c} \sum_{j=1}^{Z}S_{ij},\;\;if\;i=j,\\ 0,\;\;otherwise, \end{array}\right.$$

where

$\sum_{j=1}^{Z}S_{ij}$ = the summation of the $i^{th}$ row of matrix S, which is the number of edges connected to node i, also known as the degree of the node.

Next, the MWL matrix (L) is computed by taking the difference between the Degree matrix (D) and weighted adjacency matrix (A), as shown in Equation (5). The elements of matrix L are calculated using Equation (6). This modification helps the MWL matrix remain a strictly diagonally dominant matrix and ensures Positive Semi-Definite (PSD) properties. The final size of the MWL matrix is P × N × N. For instance, the MWL matrix L of the sample image 4th patch in Figure 2 is strictly diagonally dominant. In this matrix, the absolute summation of off-diagonal values in each row is less than 3, which corresponds to the diagonal values of L.

(5) L=D−A

(6) $$L_{ij}=\left\{\begin{array}{c} D_{ii},\;\;if\;i=j,\\ {-A}_{ij},\;if\;i\neq j, \end{array}\right.$$

#### 2.2.3. Gershgorin Circle Feature Extraction

The GC theorem estimates the eigenvalue inclusion for a square matrix in the complex plane [14]. The GC theorem states that all the eigenvalues of the square matrix are included in the union GC or Gershgorin disks. Each L matrix eigenvalue inclusion consists of radius vector $R=[r_{1}\left(L\right),r_{2}\left(L\right),\ldots ,r_{n}\left(L\right)]$ and center vector $C=[c_{1}\left(L\right),c_{2}\left(L\right),\ldots ,c\left(L\right)]$. Each GC radius and center vector of a MWL is represented as feature reduction. The elements of vector R and C are calculated according to Equations (7) and (8), respectively. The estimated radius of each GC is obtained by $i^{th}$ row absolute summation of off-diagonal values of the square matrix L denoted as $r_{i}\left(L\right)$. The center of each GC is calculated by taking the $i^{th}$ row diagonal values of square matrix L denoted as $c_{i}\left(L\right)$. Furthermore, in Figure 2, the representation of GC features can be illustrated for the 4th patch of the sample image.

(7) $$r_{i}\left(L\right)=\sum_{j\in V\backslash \{i\}}\left|L_{ij}\right|,\forall i\in V$$

(8) $$c_{i}\left(L\right)=L_{ii},\;\forall i\in V$$

where

V = Set of all nodes in the graph, with $V=\left\{\left.1,2,\ldots ,n\right\}\right.$.

$L_{ij}$ = the element of the MWL matrix at the $i^{th}$ row and $j^{th}$ column.

$L_{ii}$ = diagonal entry of MWL matrix for $i^{th}$ node.

Additionally, due to the MWL matrix being strictly diagonally dominant, all GC features lie on the real axis of the Cartesian plane [19]. Moreover, the $r_{i}\left(L\right)$ also displays a square matrix’s estimated lower and higher bounds of eigenvalues. Finally, the MWL matrix with P × N × N is reduced to the GC features with a P × N × 2, which is equivalent to P × {$r_{i}\left(L\right)\}\times {\{c}_{i}\left(L\right)\}$ matrix size.

#### 2.2.4. Classification

**Figure 3:**
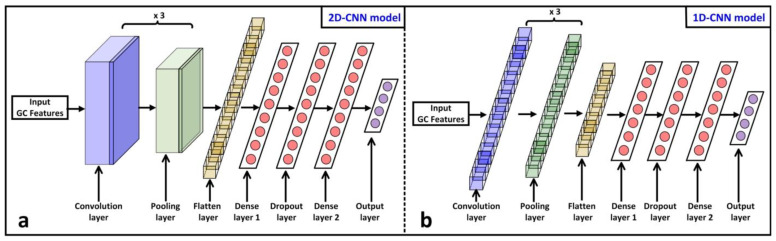
Representation of deep-learning architectures utilized in this study for feature classification. (**a**) 2D-CNN model. (**b**) 1D-CNN model.

The GCFE algorithm performance was evaluated using the 1D and 2D-CNN models for feature extraction and classification. Figure 3 shows the complete architecture for both deep-learning models [27]. The model architecture was mostly similar for all the experiments, besides a few internal layer settings, such as kernel or padding size, which were modified. In the 2D-CNN model (Figure 3a), each convolution layer’s kernel size is set to (1 × 3) for GCFE classification and (3 × 3) for other methods that were used for comparison, as shown in Table 2. Similarly, each pooling layer’s kernel is set to (1 × 2) size for the GC feature classification, while (2 × 2) is used for other methods. Since the GCFE method results in two vectors, R and C, for each patch, the kernel sizes for convolution and pooling layers were changed, as shown in Figure 2. In addition, both vectors for all individual patches of a single image are stacked up in sequence. In the 1D-CNN model (Figure 3b), all kernel and padding sizes of each convolution layer, as well as the pooling layer, are kept the same.

Initially, the GC features are fed into the input layer. The input layer for 2D-CNN was structured as (batch size, (P × {$r_{i}\left(L\right)\}$), (P × ${\{c}_{i}\left(L\right)\}$), channels), such as (1000, (1 × 784), (1 × 784), 1). For the 1D-CNN, the input layer was organized as (batch size, (P × {$r_{i}\left(L\right)\}\times {\{c}_{i}\left(L\right)\}$), channels), e.g., (1000, (1 × 784 × 784), 1). Following the input layer, the data proceed into a convolution layer. Each convolution layer for both models is configured with 32 filters, also known as a feature map. The feature maps extract different patterns from input data while training the deep-learning model. Each filter slides convoluted with input data to produce a feature map, to capture spatial hierarchies. After each convolution layer, the ReLU (Rectified Linear Unit) activation function is applied. The ReLU helps to handle the vanishing gradient problem by introducing nonlinearity to the model. After ReLU, the data are passed to the pooling layer. Each pooling layer extracts the dominant spatial features from feature maps and reduces the size of the feature map. The “average pooling” method is employed on pooling layers in both models, which calculate the average value for each patch on the feature map. Furthermore, each model has two more sets of convolution layer + ReLU + pooling layer sequentially connected. After the last pooling layer of the model, the data are transformed into the 1D vector using the flattening layer. It helps connect the convolution part of the model to the upcoming fully connected layer.

The Fully Connected Neural Network is built by connecting two dense layers in sequence with a Dropout Layer. In a Fully Connected Neural Network, each layer’s artificial neurons are fully interconnected with all artificial neurons of the next dense layer. Each dense layer has 512 artificial neurons and utilizes a nonlinear ReLU activation function. The Dropout layer is set to 0.1 (equivalent to 10%) for model overfitting regularization. In the Dropout layer, the fractions of neurons are randomly dropped out (i.e., setting to zero) during the training of the model. Finally, the output layer is interconnected with dense layer 2. The SoftMax activation function is utilized for the output layer. The SoftMax function normalizes the input data into a probability distribution over the target classes where the sum of all probabilities equals one. The number of neurons in the output layer varies according to the number of classes in the datasets. The “SpareCategoricalCrossentropy” and “Adam” are used as “loss functions” and optimizers for both CNN models. The detailed mathematical description of CNN can be found in [23].

## 3. Results and Discussion

This study compares the proposed method with seven feature reduction methods and one non-feature reduction algorithm with identical CNN classification architecture. In addition, while keeping the same environment all over the experiment, the true performance of the proposed method is evaluated. All the experiments were executed on a university supercomputer server, which was configured with 24 Core and 24 GB memory per core. The cross-validation technique is used to validate the model’s performance. Table 2 displays a comparative analysis of the proposed method in three different ways with different datasets, graph types, classification architecture, and assessment metrics.

**Figure 4:**
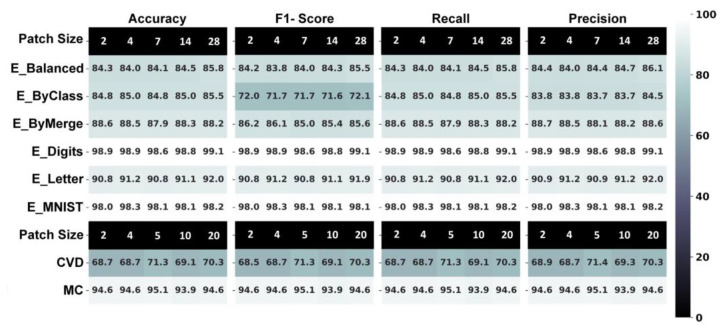
GCFE performance metric for all datasets with different image patch sizes and 2D-grid graph classified using 2D CNN.

In the first approach, the GCFE performance was examined on different patch sizes of images using 2D CNN, as shown in Figure 4. All the GCFE experiments in Figure 4 were based on a 2D grid graph. In this experiment, the datasets were split into training, validation, and testing, with ratios of 70%, 15%, and 15%, respectively. The CNN models were trained with 10 epochs. The EMNIST datasets were tested with 2, 4, 7, 14, and 28 patch sizes, while the MC and CVD datasets were experimented with 2, 4, 5, 10, and 20 patch sizes. Also, from Figure 4, it can be seen that the GCFE performance across different image patch sizes remains almost consistent, with an average standard deviation accuracy of ±0.4475. The average GCFE accuracy performance along with standard deviation (SD) for each dataset are 84.53 ± 0.714, 85.01 ± 0.281, 88.30 ± 0.269, 98.86 ± 0.154, 91.18 ± 0.473, 98.12 ± 0.124, 94.54 ± 0.404, and 69.62 ± 0.157 for E_Balanced, E_ByClass, E_ByMerge, E_Digits, E_Letter, E_MNIST, MC, and CVD, respectively (from Figure 4). Besides each model’s accuracy, other evaluating metrics, such as the F1 score, Recall, and Precision, were also computed, which is illustrated in Figure 4. Notably, the CVD dataset demonstrated lower performance, which can be attributed to its inherent characteristics—specifically, the significant presence of extraneous objects in the background as compared to the target foreground objects (cats or dogs). Further analysis revealed that in approach 2, the CVD dataset consistently showed lower accuracy across all feature extraction methods compared to other datasets.

In the second approach, additional experiments were conducted to compare the feature extraction algorithms with different graph types, their accuracy, and computational time presented in Table 3. In addition, all the experiments in Table 3 are performed on datasets with balanced distribution classes, which were E_Balanced, MC, and CVD datasets. Furthermore, the number of epochs and ratios of split datasets were kept similar to approach 1. However, due to memory resource limitations, only smaller image patch sizes were selected. For image-to-graph transformation, GCFE and the other experiments utilize two different graph types: 2D-grid and pairwise graphs, respectively. Each graph type had seven different experiments for comparison: GCFE (2D-CNN); Laplacian (2D-CNN); GCFE (1D-CNN); I-PCA (1D-CNN); kernel-PCA (1D-CNN); spectral embedding (1D-CNN); and Raw Image (2D-CNN), accordingly. In Table 3, the letter “P” in the dataset name represents the patch size. For instance, “E_balanced_P2” means an E_Balanced dataset with patch size 2. In Table 3, the experiment titled “Raw Image” is conducted to provide an approximate performance assessment for each dataset patch size.

Furthermore, similar to the findings in Figure 4 regarding GCFE performance across different image patch sizes, Table 3 also indicates similar accuracy performance trends for the GCFE method using the 2D-grid graph and the 1D-CNN model. In addition, for the E_Balanced patch size experiments, the accuracy deviated by merely ±0.4497 SD. In contrast, the accuracy performance for other feature extraction methods like PCA, kernel-PCA, and spectral embedding increased with increasing patch size. For instance, the accuracy increases from 76.468% to 78.223% with SD ± 1.0044 for I-PCA, 78.138% to 80.580% with SD ± 1.2467 for kernel-PCA, and 75.787% to 77.755% with SD ± 1.0166 for spectral embedding as the patch size varied from 2 and 4 to 7. Also, similar trends can be observed for the pairwise graph type for the E_Balanced dataset.

**Figure 5:**
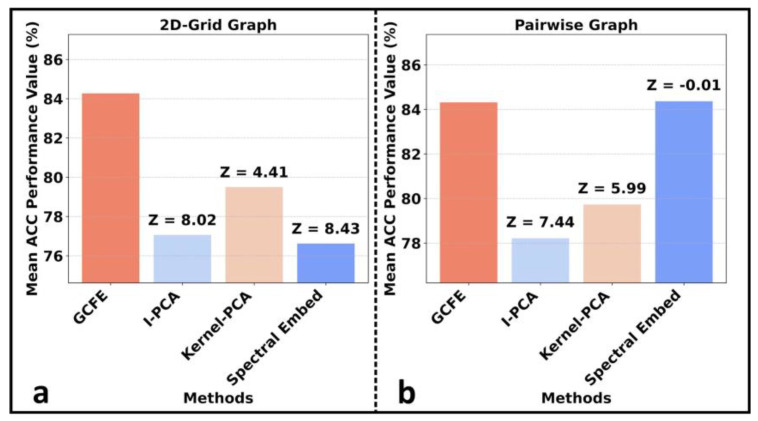
Comparison of mean ACC performance across feature extraction methods along with average Z-score for two graph types on E_Balanced dataset. (**a**) with 2D-grid graph. (**b**) with pairwise graph.

In the “Laplacian” experiment, the standard weighted Laplacian matrix was constructed and applied either as input data for various feature extraction methods or fed directly into the classification model. The feature extraction algorithms, such as I-PCA, kernel-PCA, and spectral embedding, were configured to produce the same quantity of features as the GCFE output. This configuration ensures a real performance comparison between the methods. For a detailed examination, configurations and associated code for all methods are available at Supplementary Materials [28]. Figure 5 shows each feature extraction method’s mean accuracy (ACC), utilizing both graph and 1D-CNN. It also displays the average Z-score between GCFE and other individual feature extraction methods. Both average, ACC, and average Z-score were computed for E_Balanced and patches P2, P4, and P7, accordingly. As can be seen in Figure 5a, b, GCFE consistently outperforms all other methods across both graph types, as indicated by its predominantly positive Z-score values. The only exception is the spectral embedding with the pairwise graph, which has a Z-score of −0.01 in Figure 5b. Additionally, the GCFE method ACC on CVD_P2 and MC_P2 is also much higher compared to other feature extraction methods for both graph types. For instance, when considering the 2D-grid graph type, the percentage difference between GCFE (1D-CNN) and I-PCA for CVD_P2 and MC_P2 was 5.16% and 37.04%, respectively.

Besides the comparison of feature extraction accuracy performance, the computational time for the feature extraction algorithm is an important criterion. Table 3 also presents the computation time needed to perform feature extraction on all datasets. The presented times were in seconds. Note that Table 3 has two types of time notation: “t” and “t*”. The “t” represents the time to compute all dataset instances simultaneously, while “t*” indicates the time taken when processing the dataset in smaller batches, obtaining their reduced features and subsequently implementing the dot product on the remaining instances. The GCFE computed 131,600 E_Balanced instances for a 2D-grid graph in approximately 6 s (actual 6.044 s), 6 s (actual 5.868 s) and 16 s with patch sizes 2, 4, and 7, respectively, and took 32 s and 36 s for 27,558 MC and 25,000 CVD instances with patch size 2. For the pairwise graph, it processed the same E_Balanced instances in approximately 5 s (actual 5.765 s), 5 s (actual 5.714 s), and 8 s and took 27 s and 33 s for the MC and CVD instances, all with patch size 2. Thus, the computational time for both graph types of GCFE is much lower compared to other methods, such as I-PCA, kernel-PCA, and spectral embedding. However, considering the small batch and “*dot-product*” method for feature extraction, the computation time for a spectral embedding patch size of 2 (both graph types) was lower than the GCFE. Still, with the increasing patch size of the image, the small batch and dot-product method for computational time increased more than the GCFE method. For instance, the spectral embedding method with 2D-grid and pairwise graph—E_Balanced_P4, E_Balanced_P7, CVD_P2, and MC_P2 shown in Table 3.

**Figure 6:**
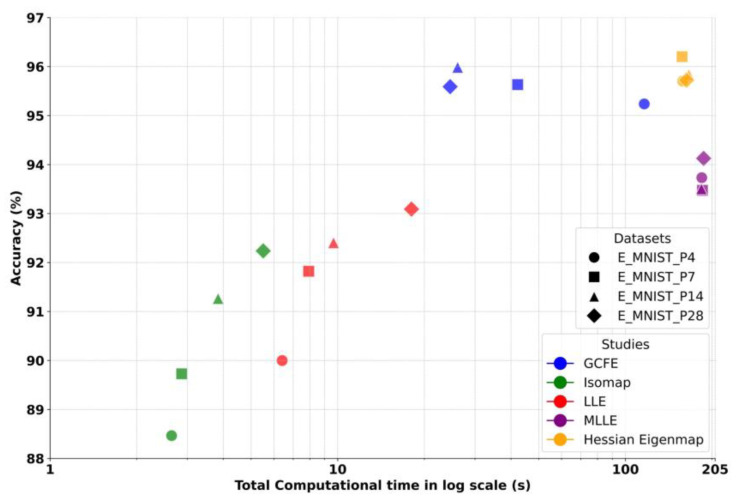
Accuracy vs. total computational time (generating graph to feature reduction) in log scale between various feature reduction methods on E_MNIST dataset with different image patch sizes.

In the third comparison approach, the GCFE was compared with additional feature reduction, which included Isomap, LLE, Modified LLE (MLLE) [29], and Hessian Eigenmap [30]. In this approach, the feature reduction methods were compared by their accuracy and total computational time (generating graph till feature reduction), as shown in Figure 6. The K-NN graph type is utilized to convert images to graphs. During these experiments, the “K” value for the graph was selected to match the image’s patch size for LLE, MLLE, and Isomap, while for the Hessian Eigenmap, K was set to 300. A total of 300 components (no. of reduced features) were chosen for the LLE, MLLE, and Isomap methods and 20 components for the Hessian Eigenmap method. Similarly, in approach 2, the LLE, MLLE, Isomap, and Hessian Eigenmap methods were applied to a small subset of the dataset comprising 1000 samples (100 samples for each E_MNIST class). Later, the rest of the datasets are transformed into reduced features by the dot product between the reduced feature of the small subset and the entire dataset. In Figure 6, similar trends were noticed in approach 2, where the accuracy performance of LLE, MLLE, and Isomap decreased with a decrease in the patch size of the image, while the GCFE and Hessian Eigenmap did not have a major variation in accuracy performance. Moreover, the GCFE outperformed the LLE, MLLE, and Isomap in classification accuracy. The GCFE and Hessian Eigenmap methods showed only minor differences in accuracy performance. However, Figure 6 indicates that the Hessian Eigenmap had a higher computational time compared to GCFE. Additionally, LLE and MLLE had lower computational times than GCFE due to the smaller dataset subset selected for feature reduction.

**Figure 7:**
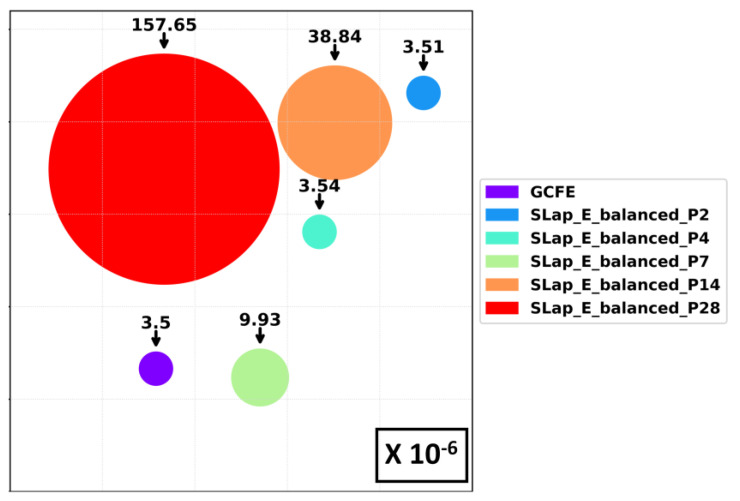
Number of training parameters (scaled by a factor of $10^{-6}$) of 2D CNN model for GCFE and standard Laplacian (SLap) features.

Figure 7 illustrates the number of training parameters of the 2D-CNN model for different patch sizes of the E_Balanced dataset—standard Laplacian (2D-CNN) features and GCFE (2D-CNN) in approach 2. In Figure 7, each circle represents the number of training parameters, which are scaled down to $10^{-6}$. The number of required training parameters for all standard Laplacian features is 3.51 for patch 2, 3.54 for patch 4, 9.93 for patch 7, 38.84 for patch 14, and 157.65 for patch 28. Comparatively, the GCFE has only 3.5 training parameters with an average percentage difference of only 0.684% (for 2D-grid type) and 0.952% (for pairwise type) compared to the standard weighted Laplacian method. Note that the number of training parameters for GCFE will remain the same for different patch sizes.

Additionally, the results demonstrate that the GCFE method offers robust and reliable feature extraction, with minimal variability in performance as indicated by its low SD and higher ACC across different datasets and graph types. This consistency is crucial for applications in computer vision, where the precision of feature extraction can significantly impact the accuracy of subsequent tasks such as image classification. In this study, the GCFE method exhibited an average SD of 0.3202 using the 2D-grid graph type across all datasets and an SD of 0.305 using the K-NN graph type on the E_MNIST dataset. These SD results demonstrate the method’s consistent ACC performance across different image patch sizes, reducing uncertainty in the GCFE method’s performance.

## 4. Conclusions

This work demonstrated a new feature extraction method for a weighted Laplacian matrix using the GC theorem. The proposed GCFE method was compared against various feature extraction algorithms while utilizing an identical CNN architecture. With only a few exceptions, the GCFE method outperformed other feature extraction methods, having a positive Z-score on both graph types. In addition, the performance accuracy of GCFE was consistent with different patch sizes of images. The GCFE method also required a much lower number of training parameters for classification models without any substantial change in accuracy compared to the standard weighted Laplacian method. This makes GCFE a good alternative solution for resource-constrained environments. Beyond accuracy, the GCFE method is computationally time efficient compared to other methods. However, it is essential to consider that GCFE is an irreversible feature reduction technique. This means that once features are extracted, they cannot be transformed back to their original state. This method is constrained by the inherent limitation that it extracts a fixed number of features from any given image, which has dimensions P × M × N, ultimately producing a reduced output of P × N × 2. Unlike parametric methods such as Principal Component Analysis (PCA), which allow for the adjustable output of dimensionality through parameters, GCFE is a non-parametric approach. Despite this limitation in feature extraction capacity, the performance of the GCFE method is not compromised. This is because the GC theorem effectively estimates the complete inclusion of all eigenvalues for a given image, ensuring that the essential features are retained even with reduced dimensionality.

In the future, the proposed GCFE method could be applied to diverse fields, such as biomedical signal analysis. Additionally, enhancing the method by incorporating more mathematically precise eigenvalue inclusion techniques could further improve the classification accuracy of the reduced features.

## Figures and Tables

**Table 1:** Different properties of dataset.

Datasets	Instances	Classes	Type	Distribution of Classes
EMNIST				
E_Balanced	131,600	47	1D	Balanced
E_ByClass	814,255	62	1D	Imbalanced
E_ByMerge	814,255	47	1D	Imbalanced
E_Digits	280,000	10	1D	Imbalanced
E_Letter	145,600	26	1D	Imbalanced
E_MNIST	70,000	10	1D	Imbalanced
CVD	25,000	2	3D	Balanced
MC	27,558	2	3D	Balanced

**Table 2:** Overview of GCFE comparison approaches across diverse datasets and graph structures using different classification architectures and performance metrics.

Approach No.	Dataset	Type of Comparison	Type of Graph	Classification Architecture	Performance Metric
1	E_Balanced, E_ByClass, E_ByMerge, E_Digits, E_Letter, E_MNIST, CVD, MC	Comparison between GCFE of all datasets with different image patch sizes.	2D-Grid	2D-CNN	Accuracy
2	E_Balanced, CVD, MC	Comparison between GCFE, Laplacian, I-PCA, Kernel-PCA, and spectral embedding with different image patch sizes.	2D-Grid, pairwise	2D-CNN, 1D-CNN	Accuracy, Z-Score
3	E_MNIST	Comparison between GCFE, Isomap, LLE, MLLE, and Hessian Eigenmap with different image patch sizes.	K-NN	1D-CNN	Accuracy

**Table 3:** Comparison of proposed GCFE with other methods by measuring accuracy performance and computational time.

Datasets	GCFE (2D CNN)	Laplacian (2D CNN)	GCFE (1D CNN)	I-PCA	Kernel-PCA (RBF)	Spectral Embed.	Raw Image
Graph type—2D-Grid							
E_Balanced_P2	84.329 t = ≈6	84.553	83.797 t = ≈6	76.468 t = ≈225	78.138 t* = ≈12	75.787 t* = ≈3	86.617
E_Balanced_P4	83.978 t = ≈6	85.010	84.691 t = ≈6	76.499 t = ≈926	79.776 t* = ≈107	76.329 t* = ≈54	86.117
E_Balanced_P7	84.117 t = ≈16	84.595	84.329 t = ≈16	78.223 t = ≈2010	80.585 t* = ≈1163	77.755 t* = ≈1136	85.659
CVD_P2	68.681 t = ≈32	71.518	66.045 t = ≈32	62.722 t = ≈10,475	~	61.318 t* = ≈1182	70.773
MC_P2	94.581 t = ≈38	93.783	92.646 t = ≈38	63.691 t = ≈6944	~	62.796 t* = ≈1256	92.186
Graph type—Pairwise							
E_Balanced_P2	84.744 t = ≈5	85.372	84.989 t = ≈5	78.595 t = ≈194	79.287 t* = ≈11	77.638 t* = ≈1	86.617
E_Balanced_P4	84.276 t = ≈5	85.255	84.148 t = ≈5	77.297 t = ≈917	80.553 t* = ≈111	95.86 t* = ≈55	86.117
E_Balanced_P7	83.237 t = ≈8	84.074	83.808 t = ≈8	78.755 t = ≈1912	79.351 t* = ≈1122	79.595 t* = ≈1182	85.659
CVD_P2	69.684 t = ≈27	70.773	69.025 t = ≈27	~	~	63.954 t* = ≈1337	70.773
MC_P2	92.597 t = ≈33	92.938	92.670 t = ≈33	~	~	63.570 t* = ≈1407	92.186

“~” = Out of Memory; “t” = total time computational for all samples; “t*” = total time computational for all samples by extracting a small bundle of samples and the rest of samples dot product (all computational time in seconds).

## Data Availability

All the image data used to support the findings of this study are from previously reported studies and datasets. These prior studies and datasets are cited at relevant places within the text as references [24,25,26].

## Supplementary Materials

All codes related to this work are available at https://github.com/sahaj432/Image_GCFE.git, accessed on 12 February 2024.
